# Detection of virulence genes of *Staphylococcus aureus* isolated from raw beef for retail sale in the markets of Ulaanbaatar city, Mongolia

**DOI:** 10.1186/s12866-023-03122-2

**Published:** 2023-11-29

**Authors:** Amgalanzaya Dorjgochoo, Anujin Batbayar, Altansukh Tsend-Ayush, Otgontsetseg Erdenebayar, Bayarlakh Byambadorj, Sarantuya Jav, Munkhdelger Yandag

**Affiliations:** 1 Department of Biomedicine, Etugen University, Ulaanbaatar, Mongolia; 2 Department of Molecular Biology and Genetics, School of Biomedicine, Mongolian National University of Medical Sciences, Ulaanbaatar, Mongolia; 3 Global Leadership University, Ulaanbaatar, Mongolia

**Keywords:** Foodborne infection, *S. aureus*, Antibiotic resistance, Raw meat, Virulence genes, *sea*, *sed*, *tsst*, *eta*, *etb*, *mecA*

## Abstract

**Background:**

*Staphylococcus aureus (S. aureus)* is a highly virulent pathogen that causes food-borne illness, food poisoning, skin and soft tissue infections, abscesses, mastitis, and bacteremia. It is common for meat and meat products to become contaminated with *S. aureus* due to dirty hands, food storage conditions, food production processes, and unhygienic conditions, causing food poisoning. Therefore, we aimed to isolate *S. aureus* strain from the raw beef and reveal virulence genes and antibiotic resistance profile from isolated *S. aureus* strains.

**Methods:**

In this study, 100 samples of raw beef were collected from 4 major market stalls in Ulaanbaatar city, Mongolia. *S. aureus* was detected according to the ISO 6888–1:2021 standard, and the *nucA* gene encoding the species-specific thermonuclease was amplified and confirmed by polymerase chain reaction (PCR). In the strains of *S. aureus* isolated from the samples, the genes encoding the virulence factors including *sea, sed, tsst, eta, etb,* and *mecA* were amplified by multiplex PCR. These genes are encoded staphylococcal enterotoxin A, enterotoxin D, toxic shock syndrome toxin, exotoxin A, exotoxin B and penicillin-binding protein PBP 2A, respectively. Antibiotic sensitivity test was performed by the Kirby–Bauer disc diffusion method. The Clinical and Laboratory Standard Institute guidelines as CLSI M100-S27 was used for analysis of the data.

**Results:**

Thirty-five percent of our samples were detected contaminated with of the *S. aureus* strains. Subsequently, antibiotic resistance was observed in the *S. aureus* contaminated samples. Among our samples, the highest rates of resistance were determined against ampicillin (97.1%), oxacillin (88.6%), and penicillin (88.6%), respectively. Three genes including *mecA, sea,* and *tsst* from six virulence genes were detected in 17% of *S. aureus* strain-contaminated samples by multiplex PCR. The *sed*, *etb* and *eta* genes were detected in the 2.9%, 11.4% and 5.7% of our samples, respectively.

**Conclusion:**

The results show that *S. aureus* related contamination is high in the raw beef for retail sale and prevalent *S. aureus* strains are resistant to all antibiotics used. Also, our results have demonstrated that there is a high risk for food poisoning caused by antibiotic resistant *S. aureus* in the raw beef and it may establish public health issues. Genes encoding for both heat-resistant and nonresistant toxicity factors were detected in the antibiotic resistant *S. aureus* strains and shown the highly pathogenic. Finally, our study is ensuring to need proper hygienic conditions during beef’s preparation and sale.

## Introduction

*Staphylococcus aureus* (*S. aureus*) is a highly virulent bacterial pathogen that causes food-borne diseases, food poisoning, skin and soft tissue infections, pneumonia, mastitis, and bacteremia [1, 2].

*S. aureus* presents abundantly in large quantities on skin and mucous membranes of humans and animals, and it is transmitted directly or indirectly from an infected person [3]. Raw and semiprocessed food products, such as meat, meat products, eggs, milk, and dairy products, are often contaminated with *S. aureus* due to dirty hands, food storage conditions, food production, and unhygienic conditions [4, 5]

In recent years, many studies on the distribution of *S. aureus* have been conducted around the world, but in our country, there are few studies on the distribution of *S. aureus* in food products. Shi Wu and Jiahu Huang et al. have reported that 35% of the samples collected 39 cities from 2011 to 2016 in China was positive for *S. aureus*, and 51% of the highly contaminated samples were raw meat [6]. According to a 2017 study by Tang, 27.9% of raw meat samples for retail sale in the United States and 68% in Denmark were contaminated with *S. aureus* [7]. Mongolia ranks 10^th^ in the world in per capita meat consumption, with 108.8 kg of meat consumed per person per year, which is 2.7 times higher than the average world consumption. According to the data released by the National Statistics Committee of Mongolia, 69.3% of meat products are consumed including 2.3% of pork products, 0.4% of poultry, 15% of sausages and canned meat, and 13% of seafood [8].

Staphylococcal food poisoning is a common food-borne illness caused by *S. aureus* enterotoxin contamination. Because *S. aureus* contains a wide range of toxic factors, food poisoning and foodborne infections caused by *staphylococcus* are a major public health problem [9]. In particular, staphylococcal enterotoxin, toxic shock syndrome toxin, and exotoxin are resistant to high temperatures and acidic environments, so they cause food poisoning without being broken down by digestive enzymes [10]. Strains containing heat-resistant virulence factors can also contaminate ready-to-eat food and infect humans when hygienic safety is lost [11].

The imprudent and indiscriminate usage of antibiotics in public and veterinary clinical practices has led to the development of multiple drug-resistant (MDR) pathogens. Commonly practiced antibiotics for treatment i.e. beta-lactams, macrolides, lincosamides, streptogramins, and fluoroquinolones are facing resistance due to their undue and persistent usage in animals. The number of effective antibiotics against MDR pathogens is rapidly declining and the advent of new antibiotics in clinical practice requires a prolonged time and has monetary challenges as well [12].

As *S. aureus* has developed and adapted its resistance mechanism to antibiotics, for instance, the strains resistant not only to penicillin and methicillin but also to the linezolid and daptomycin [13–15].

One public health concern is that antibiotic resistance in bacteria continues to increase worldwide. The imprudent and indiscriminate usage of antibiotics in public and veterinary clinical practices has led to the development of MDR pathogens. For instance beta-lactam antibiotics are commonly used in the treatment of staphylococcal infections, but the number of strains of *S. aureus* that secrete beta-lactamase and are resistant to lactam antibiotics is increasing [16]. In 1960, methicillin, a new antibiotic in the penicillin group, was discovered and used effectively in the treatment of *S. aureus* infections, but the incorporation of outside DNA encoding the *mecA* gene led to methicillin resistance, and thus the therapeutic effect of all beta-lactam antibiotics decreased [17]. In the Netherlands and Canada, 20–40% of all *S. aureus* strains detected in pork were identified as methicillin-resistant *S. aureus* (MRSA) [18]. *S. aureus* has human and swine isolates that were resistant to penicillin, oxacillin, and tetracycline, and susceptible to trimethoprim-sulfamethoxazole, gentamicin, levofloxacin, moxifloxacin, linezolid, daptomycin, vancomycin and rifampin [19].

In our country, there is a high risk of food poisoning caused by *S. aureus* due to high consumption of meat, uncontrolled use of antibiotics for livestock, and insufficient hygienic environment for retail sales. This study was designed to detect *S. aureus* contamination in retail meat, and prevent from *S. aureus*-related food poisoning and provide appropriate treatment by detecting the virulence and antibiotic resistance of the pathogen. Hence, it would be a basic research to improve the hygienic environment in which meat prepared and sold, and provide warning information that the meat should be fully processed and consumed for customers.

## Materials and methods

This study was conducted using a cross-sectional design. Raw beefs are sold at retail in 4 major marketplaces, which located differently in Ulaanbaatar city. Then, 100 samples of raw beef were collected from the above-mentioned marketplaces from June to December 2021, and delivered within two hours to the microbiological laboratory of the Department of Molecular Biology and Genetics, Mongolian National University of Medical Sciences.

### Isolation of *S.aureus* from the samples

To detect the *S. aureus* from collected samples, 25 g meat samples were placed in 225 ml of peptone broth, and 1 ml of the homogenized solution was diluted by a factor of 10^3^ according to ISO 6888–1:2021. One milliliter of the diluted solution was taken and distributed evenly in a Baird-Parker (BP) selective medium environment (Biolab, Hungary). The plates were incubated at 37°C for 24 hours. After incubation, black colonies with a clear border were evaluated as suspected *S. aureus. *Up to five colonies were chosen, and PCR assay was applied for the identification of *S. aures* [3].

### Identification of *S. aureus* using PCR assay

DNA was extracted by preparing a bacterial suspension from the culture, boiling it at 100°C for 10 min, followed by sedimentation in a centrifuge for 10 min at 12000 rpm [20]. Then, DNA was checked by nanodrop spectrophotometer (Thermo fisher Scientific. LLC) for yield and purification, and used for further analysis.

The 270 kb product of the *nucA* gene encoding a species-specific thermonuclease of *S. aureus* was amplified and confirmed by PCR. A total of 0.5 μl (100 pmol/μl) of each primer (nuc-F 5′-3'GCGATTGATGGTGATACGGTT), (nuc-R 5′-3′AGCCAAGCCTTGACGAACTAAAGC), and 2 μl of DNA were prepared in the PCR master mix (Bioneer, Korea), and a total of 25 μl of solution was reacted at 95 °C for 10 min, followed by 37 cycles of 94 °C for 1 min, 55 °C for 30 s, 72 °C for 1.5 min, and 72 °C for 10 min. The amplified PCR products were detected by running a 1.5% agarose gel at 100 V for 30 min and staining with ethidium bromide for 20 min [3, 20].

### Detection of *S. aureus* virulence genes by multiplex PCR

*S. aureus* enterotoxin (*sea, sed*), toxic shock syndrome toxin (*tsst*), exotoxin (*eta, etb*) and penicillin-binding protein (*PBP 2A*) and its coding gene *mecA* were amplified in 2 sets by multiplex PCR using the primers in Table 1 [21].

**Table 1:** Primers for the amplification of staphylococcal genes

Genes	Sequences (5’-3’)	Size of amplified product (bp)	Set
*mecA*	F-ACTGCTATCCACCCTCAAAC R-CTGGTGAAGTTGTAATCTGG	163	Set 1
*sed*	F-CCAATATAGGAGAAAATAAAAG R-ATTGGTATTTTTTTTCGTTC	278	
*eta*	F-GCAGGTGTTGATTTAGCATT R-CTGGTGAAGTTGTAATCTGG	93	
*sea*	F-GGTTATCAATGTGCGGGTGG R-CGGCACTTTTTTCTCTTCGG	102	Set 2
*etb*	F-GGTTATCAATGTGCGGGTGG R-GTTTTGGTGCTTCTCTTG	226	
*tsst*	F-ACCCCTGTTCCCTTATCATC R-TTTTCAGTATTTGTTAACGCC	326	

The PCR steps were as follows: initial denaturation at 95 °C for 5 min, followed by 30 cycles of denaturation at 94 °C for 2 min, annealing at 55 °C for 2 min, extension at 72 °C for 2 min, followed by a final 7 min extension at 72 °C. The amplified PCR products were detected by running a 1.5% agarose gel at 100 V for 30 min and staining with ethidium bromide for 20 min [3, 21].

### Antibiotic susceptibility testing

The disk diffusion method was used to determine the antibiotic susceptibility of the isolates on Muller Hinton agar (Difco, Franklin Lakes, NJ, USA). Each isolate was tested for antibiotic susceptibility using a panel of the following antibiotics: ampicillin (10 μg), oxacillin (30 μg), gentamicin (10 μg), tetracycline (30 μg), chloramphenicol (50 μg), penicillin (10 μg), clindamycin (2 μg), azithromycin (15 μg) and ciprofloxacin (5 μg) (Biolab, Budapest, Hungary). The plates were incubated at 37 °C for 24 h, and the inhibitory zone diameters were measured. Observed result was compared with the criteria recommended by the Clinical and Laboratory Standards Institute guidelines (CLSI M100-S27) and interpretated. Standard *S. aureus* strain ATCC 25923 was used for validation of antibiotic discs.

### Statistical analysis

Statistical analysis of the results in this research work were calculated using SPSS 25. Statistical analysis was performed by using Fisher’s exact test, chi-square test. The level of significance was set at a *p* value of < 0.05.

## Results

### Detection of *S. aureus* contamination in the samples

**Fig. 1:**
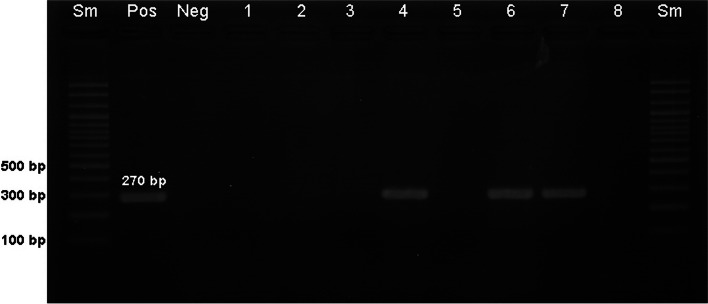
*nucA* gene identification. Note: Sm-size marker (100 bp), pos-positive control, and neg-negative control: nuclease free distilled water. Samples 4, 6, and 7 showed amplification of *nucA* gene product at 270 bp. *nucA* gene product wasn’t amplified in samples of 1, 2, 3, 5 and 8

In this study, the contamination of *S. aureus* strains were detected in 35% from total samples and validated by expression of gene *nucA* (Fig. 1).

### Determination of the antibiotic resistance of the *S. aureus* strains

**Fig. 2:**
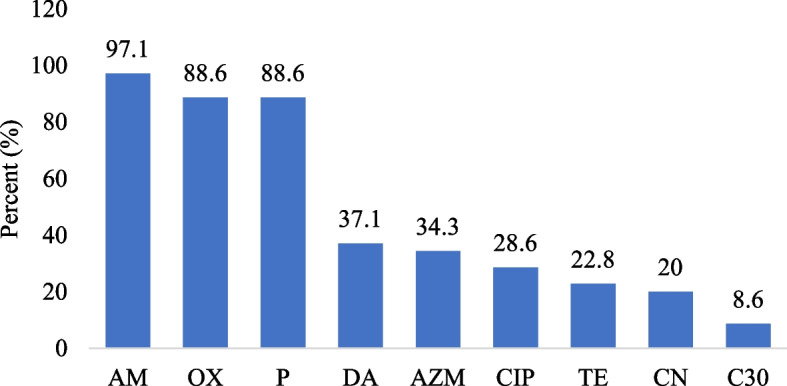
Antibiotic resistance of *S. aureus* detected in samples. Abbreviations: AM-ampicillin, OX-oxacillin, CN-gentamicin, TE-tetracycline, C30 chloramphenicol, P-penicillin, DA-clindamycin, AZM-azithromycin, CIP-ciprofloxacin

Figure 2 has shown that the highest resistance to ampicillin 97.1% (34/35), oxacillin 88.6% (31/35), and penicillin 88.6% (31/35) observed in the *S. aureus* strains. In contrast, the resistance to chloramphenicol 8.6% (3/35) was the lowest.

Forty percent (14/35) of *S. aureus* isolated from the samples were determined as resistant to more than 3 groups of antibiotics, indicating that the strains are multidrug-resistant (MDR). In our study, 3 and 4 groups of antibiotic-resistant *S. aureus* strains was identified in 11 samples, as well as 5 groups of antibiotic-resistant strains were identified in 2 samples, respectively. One strain was resistant to all 9 antibiotics of the 7 groups tested.

### Detection of virulence factor genes in *S. aureus* strains

**Fig. 3:**
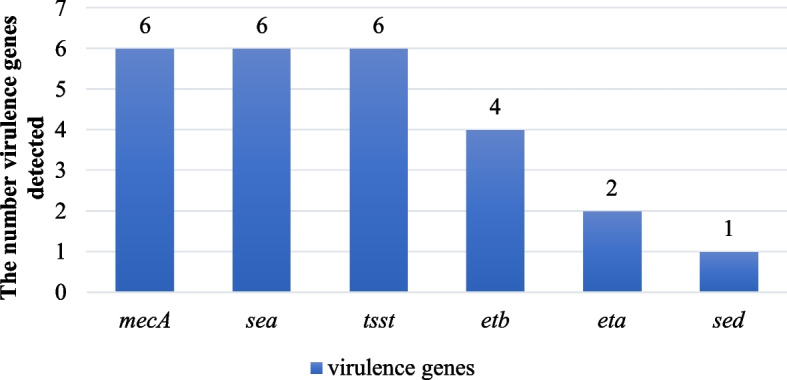
Detection of virulence genes

Six virulence genes were detected by multiplex PCR in the *S. aureus* strains isolated from meat. As shown in Fig. 3, number of three genes including encoding penicillin-binding protein (*PBP 2A*) (*mecA*), enterotoxin A (*sea*), and toxic shock syndrome toxin (*tsst*) were observed in 17.1% (6/35) of the contaminated samples. Moreover, exotoxin type A (*eta*) and exotoxin type B (*etb*) were found in 5.7% (2/35) and 11.4% (4/35), as well as enterotoxin D (*sed*) in 2.9% (1/35) among contaminated samples.

Table 2 shows the overlap of the virulence genes in the *S. aureus* strains.

**Table 2:** Detection of overlapping virulence genes in *S. aureus*

Frequency of the detected gene	Sample number (n)	Percent (%)	Detected genes
Not detected at all	18	51.4	-
1 gene	12	34.3	-
2 gene	3	8.6	*mecA, tsst*
3 gene	1	2.9	*mecA, sed, etb*
4 gene	1	2.9	*mecA, eta, etb, sea*
Total	35	100	-

Four genes such as *mecA, eta, etb, sea* were similarly identified in 14.3% (5/35) of the *S. aureus* strains, but only one gene was identified in 34.3% (12/35) of those samples. The presence of virulence genes was evaluated in both antibiotic-resistant and antibiotic-sensitive groups (Table 3). No statistically significant differences were observed when evaluating the expression of virulence genes in the antibiotic-resistant and antibiotic- susceptibility groups (*p* > 0.05).

**Table 3:** Detection of virulence genes among the antibiotic-resistant *S. aureus* strains

Antibiotic	*mecA* n (%)	*sed* n (%)	*eta* n (%)	*etb* n (%)	*sea* (%)	*tsst* n (%)	Total n
AM	6 (17.6)	1 (2.9)	1 (2.9)	3 (8.8)	5 (14.7)	6 (17.6)	34
OX	6 (19.4)	1 (3.2)	2 (6.4)	4 (12.8)	5 (16.1)	6 (19.4)	31
P	6 (19.4)	1 (3.2)	1 (3.2)	2 (6.4)	4 (12.8)	5 (16.1)	31
CN	2 (28.6)	0	0	0	1 (14.3)	3 (42.9)	7
AZM	4 (33.3)	1 (8.3)	1 (8.3)	1 (8.3)	1 (8.3)	3 (25)	12
CIP	2 (20)	0	0	0	0	2 (20)	10
TE	2 (25)	0	0	0	1 (12.5)	2 (25)	8
C30	0	0	0	0	0	1 (33.3)	3
DA	2 (15.4)	0	1 (7.7)	0	1 (7.7)	2 (15.4)	13

*Abbreviations*: *AM* ampicillin, *OX* oxacillin, *CN* gentamicin, *TE* tetracycline, *C30* chloramphenicol, *P* penicillin, *DA* clindamycin, *AZM* azithromycin, *CIP* ciprofloxacin

## Discussion

Meat and meat products are widely consumed in all countries and are rich in fat, protein, and many vitamins. In the last 50 years, following the doubling of the world's population, the consumption of meat products used in food has tripled [22].

In our country, the number of animals has reached more than 60 million, and animal products, especially meat, are used for food. We are ranked 10^th^ in the world in terms of meat consumption, with 2.7 times more meat consumed than the world average [8].

The prevalence of *S. aureus* contamination in retail meat may depend on the number of samples studied, storage conditions, retail environment, and season. In our country, the meat sold in the markets may have a relatively high rate of contamination depending on the fact that it is sold at room temperature in all seasons, is sold in the open market, and is not transported by special vehicles from the meat preparation places.

According to our study, in the markets of Ulaanbaatar, *S. aureus* was identified in 35% of the retail meat samples. There have been reported many studies around the world. In these studies, *S. aureus* was determined between 16% and 54.4% of the raw beef samples [23–27]. For instance in a study in Iran by Zeinab Torki, *S. aureus* was found in 16% of raw beef for retail sale, [23] in a study by Bizuneh Tsehaynah, 54.5%, [24] and in a study by Qianting Ou, and 29.2% [25]. In a study conducted in Japan, it was also reported that 32.8% of the raw beef samples were contaminated by *S.aureus* [26]. Moreover, a study showed that 40% of item contaminated by *S. aureus,* 77.8% and 47.2% of which had resistance at tetracycline and clindamycin, respectively [28]. As compared with other countries, level of the beef contamination was similar to the item but antibiotic resistance was higher than that of another country. By result of study conducted in Turkey, 63.54% of the meat samples was contaminated with *S. aureus* and its antibiotic resistance was observed each antibiotic for example, oxacillin 12.5%, tetracycline 26.03%, chloramphenicol 23.96%, penicillin 78.2%, clindamycin 16.66%, and azithromycin 46.87% [27].

In Mongolia, 35% of the beef samples was contaminated by *S. aureus* and its antibiotic resistance including oxacillin 88.6%, tetracycline 22.8%, chloramphenicol 8.6%, penicillin 88.6%, clindamycin 37.1%, and azithromycin 34.3% was observed in our study. Hence, the contamination of retail beef samples is considered as public health hazard in Mongolia.

Since the prevalence of bacterial infections and inflammatory diseases among the population in our country has not decreased significantly, the use of antibiotics as drug treatments has been increasing year over year, the side effects of drugs have increased, and pathogenic bacteria have become resistant to antibiotics and more virulent [29].

In Mongolia, approximately 70% of all drugs are imported, and approximately 30% of the drugs used for treatment are antibacterial agents. Additionally, more than 700 drugs are registered in the state register for animal husbandry practice, but there are risks of serious damage to human health due to the haphazard and uncontrolled use of antibiotics, the use of drugs in food before it has been completely excreted, and infection with antibiotic-resistant bacteria [29]. Common food products such as contaminated raw meat and meat products are a common way by which antibiotic-resistant bacteria spread from animals to humans [30, 31]. In other words, the improper use of antibiotics in animals can lead to high levels of antibiotic resistance in *S. aureus* strains found in meat and meat products [1]. Antibiotic resistance is likely to increase, depending on factors such as the number of animals, the improper use of antibiotics, and use of animal feed containing antibiotics.

Uncontrolled large-scale use of antibiotics is the basis for the emergence of MDR strains, and MDR *S. aureus* is quite common in hospital environments and on farms [32]. In a study conducted in the Federal Democratic Republic of Nepal, all 6 strains of *S. aureus* detected in meat were identified as resistant to antibiotics, especially amoxicillin and tetracycline [33, 34]. Additionally, in a study conducted by Pesavento G. in 2007, the staphylococcal strains detected were resistant to beta-lactam antibiotics such as oxacillin and cefoxitin, and 30.95% of them were identified as MDR. According to the results of this study, most of the staphylococci were resistant to beta-lactam antibiotics such as oxacillin and ciprofloxacin. It was found that 43 (89.58%) out of 48 *S. aureus* strains were highly resistant to beta lactam antibiotics [35]. Forty percent (14/35) of the *S. aureus* strains detected in the raw meat samples included in our study were MDR, while 1 strain was resistant to all 9 antibiotics in all of the 7 groups selected.

The prevalence of MRSA in China and Russia, which border Mongolia, is 10–50%, which is very high, showing that this type of research is necessary in our country. In our study, 17.1% (6/35) of the *S. aureus* detected in raw beef were MRSA. Over the past decade, MRSA strains have become serious pathogens that are resistant to antibacterial therapy and have spread to many regions of the world. Therefore, the rapid detection and diagnosis of MRSA is important to improve treatment outcomes, prevent the spread of infection, and reduce the risk of patient mortality.

Staphylococcal enterotoxins are the most important cause of foodborne illness [34]. Enterotoxins are highly stable toxins that are resistant to proteolytic enzymes such as pepsin, trypsin, and chymotrypsin. The heat resistance of enterotoxins vary depending on the pH and salt concentration of the environment, but on average, these toxins can withstand an environment at 100 °C for 30 min [36].

When meat contaminated with *S. aureus* is undercooked or stored at inappropriate temperatures, enterotoxins accumulate and cause staphylococcal food poisoning. In a study conducted in Taiwan from 2001 to 2003, *tsst* (59.1%), *sea* (29.2%), and *sed* (2%) were identified in 147 of the *S. aureus* strains found in patients that were associated with staphylococcal food poisoning outbreaks [37]. According to Sarrafzadeh's study, more than 50% of food poisoning caused by staphylococci was caused by enterotoxin A [38].

In our study, *tsst* was found in 17.1% of the samples, *sea* in 17.1%, and *sed* in 2.9%, which indicates the risk of food poisoning caused by *S. aureus* enterotoxin in our country. According to Hoveida L 's study, enterotoxins were found in 20.5% of the meat samples in which *S. aureus* was detected, and the virulence genes *sec* (19%), *sea* (9.5%), and *tsst* (3.5%) were identified [39]. In the study by Manisha in 2000, 24.3% of the *S. aureus* were positive for *tsst,* and 19.6% were positive for *sea* [40]. According to our study, enterotoxin A (*sea*) and toxic shock syndrome toxin (*tsst*) were each present in 17.1% (6/35) of the samples, exotoxin A was in 5.7% (2/35), and type b was in 11.4% (4/35), while enterotoxin D (*sed*) was detected in 2.9% (1/35), which is similar to the results of previous researchers.

Identifying the comparison of the virulence genes detection between antibiotic resistant and susceptibility strain groups, there were no statistically significant differences between the two groups in this study. Similarly, the toxin or virulence genes were tested in the resistant strains and it has not been identified in half of the resistant strains [41]. The study of virulence genes differences between MRSA and Methicillin-susceptible *S. aureus* (MSSA) reported that the virulence genes such as *sea* gene were higher in MRSA isolates, but *tsst* genes were not statistically significant difference both groups [42]. On the other hand, the prevalence of *sed* and *tsst* genes was significantly higher in MRSA than MSSA isolates by the study of comparative analysis of the prevalence of virulence genes between MRSA and MSSA isolates using the Chi-square and Fisher’s exact test [43]. The difference of previous and study results might depend on the isolated strain, its virulence, sample type and size for isolating *S. aureus* and geographical areas.

Food contaminated with highly toxic and antibiotic-resistant *S. aureus*, especially MRSA, can pose a serious threat to public health. An effective reduction of staphylococcal contamination levels could be achieved by improving sanitation and hygiene procedures. Therefore, there is an urgent need to develop methods and strategies to control hygiene when handling retail meat, prevent bacterial contamination, and detect contamination quickly.

Also, the contact between processed foods and unprocessed foods must be avoided. In order to ensure food safety, there is a need to expand research on detecting virulence factors responsible for food poisoning and antibiotic resistance. Prudent use of antibiotics in veterinary medicine is recommended and also education on the proper use of antibiotics should be prioritized for livestock farmers.

## Conclusion

Our study concluded that *S. aureus* contamination is high in raw meat for retail sale, and these strains are resistant to antibiotics. This contamination is a high risk of food poisoning and the possibility of complications in its treatment. In our study, *S. aureus* strains isolated from meat contain a number of genes that are encoding heat-resistant and nonresistant virulence factors and subsequently, these strains are highly pathogenic. According to our study, the proper hygienic condition is to needed for meat preparation and sale.

### Limitation of the study

Due to limitations of the funding of the research, 4 retail markets, limited samples and selective bacterial strains were included in our study. We plan to conduct further study in detail.

## Data Availability

All data generated or analyzed during this study are included in this published article.
